# Vaccination-related *Mycobacterium bovis* BCG Infection

**DOI:** 10.3201/eid1205.050107

**Published:** 2006-05

**Authors:** Anna Liberek, Maria Korzon, Ewa Bernatowska, Magdalena Kurenko-Deptuch, Marlena Rytlewska

**Affiliations:** * Medical University, Gdańsk, Poland;; † Children's Memorial Health Institute, Warsaw, Poland

**Keywords:** tuberculosis, TB, bacillus Calmette-Guerin, BCG, Mycobacterium bovis BCG Infection

**To the Editor:** The high prevalence of tuberculosis (TB) underlines the important role of BCG (bacillus Calmette-Guérin) immunization. The vaccine, however, is not free from complications, which could be local or disseminated. Disseminated BCG infection as a result of TB vaccination is a rare complication with an incidence of 0.06 to 1.56 cases per million vaccinations; it occurs exclusively in patients with immune deficits. However, in these cases, the prognosis is unfavorable; up to 70% of patients die, despite intensive antituberculous treatment (*1**–**4*).

A 4-month-old-girl exhibited enlargement of left axillary lymph nodes during a 1.5-month period. She was the second child of healthy parents, with no family history of genetic disorders or TB. She was vaccinated according to the regimen compulsory in Poland: the first dose of BCG and anti–hepatitis B virus (HBV) vaccination on the first day of life, followed by vaccination against diphtheria, tetanus, pertussis, poliomyelitis, and the second dose of anti-HBV after 6 weeks. BCG vaccination was performed intradermally in the upper part of left arm by administration of 0.1 mL Brazilian Moreau strain (Biomed, Lublin, Poland).

On hospital admission, the patient was in reasonably good condition but pale, with grossly enlarged, adjacent left axillary lymph nodes and hepatosplenomegaly. Laboratory tests showed anemia, thrombocytopenia, elevated transaminase activity, a high C-reactive protein level, and high level of immunoglobulin M (IgM) class anti-cytomegalovirus (CMV) reactive antibodies.

Based on clinical manifestations and biochemical and serologic signs, CMV infection was suspected. The patient was administered a 14-day regimen of ganciclovir (10 mg/kg/day); results of liver function tests and blood count normalized, and hepatosplenomegaly decreased. However, the lymph nodes continued to enlarge, and diagnostic excision and bone marrow aspiration were performed to exclude a neoplastic process. A histopathologic image of the excised lymph nodes showed caseating granulomas, and tuberculous lymphadenitis was suggested (Figure).

**Figure:**
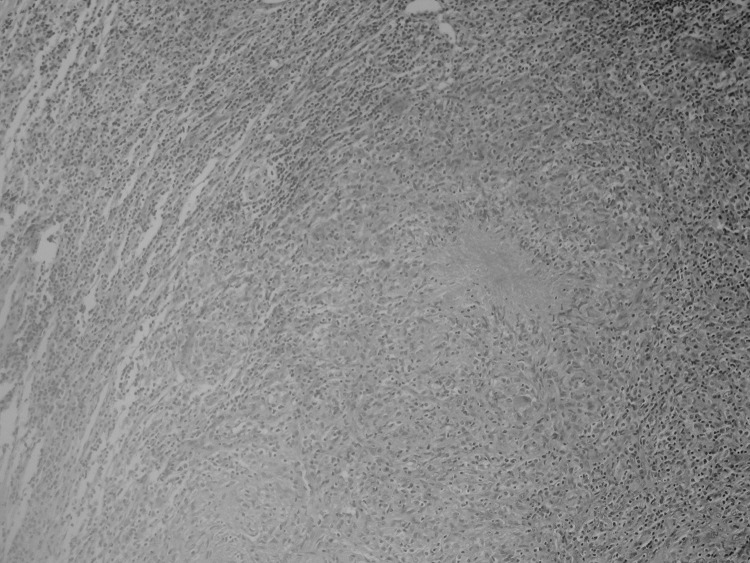
Digitally processed hematoxylin-eosin staining of the excised lymph nodes, showing caseating, tuberculosislike granulomas (original magnification ×100).

At that time, a diagnosis of disseminated BCG infection as a complication of TB vaccination in a presumed immunocompromised patient was proposed. This idea was based on suggestive lymph node pathology, which showed caseating granulomas, a history of TB vaccination, and the exclusion of other pathologic changes. Flow cytometry measurements showed abnormally low expression of the α chain of the interferon (IFN)-γ receptor on peripheral blood lymphocytes. Only 20% lymphocytes expressed CD 119 (IFN-γ receptor outer subunit R1).

Three-drug anti-tuberculous therapy (with rifampin, isoniazid, and streptomycin) was introduced despite chest and bone radiographs that were negative for infection, no abnormalities found on funduscopy, and negative results of Ziehl-Neelsen staining of lymph node tissue. Despite this therapy, the child's condition worsened; she exhibited a high temperature, hemolysis, and progressive neutropenia, thrombocytopenia, cholestasis, and renal failure. Uncontrolled sepsis developed, and she died.

At postmortem examination, the diagnosis of disseminated BGC infection was made on the basis of multiple TB-like granulomas in the lungs, lymph nodes, meninges, liver, spleen, and kidneys. However, direct microbiologic confirmation of BCG infection was lacking because cultures were negative and Ziehl-Neelsen and periodic acid–Schiff staining did not show acid-fast bacilli, other bacteria, or fungi in these specimens.

This case represents a rare complication of antituberculous vaccination, that is a progressive, disseminated BCG infection in a patient with deficiency of IFN-γ receptor. Concomitant CMV infection was diagnosed by positive IgM antibody response. Transient response to the ganciclovir treatment made the final diagnosis of BCG infection more difficult and probably postponed implementation of the anti-TB therapy. Until now ≈100 cases have been reported in the literature, most of them in infants and young children. These patients also had clear predisposition to other severe infections with intracellular microorganisms such as atypical mycobacteria, *Salmonella* spp., *Listeria monocytogenes*, and *Leishmania* spp (*1**–**5*).

The INF-γ receptor is present on many cell types; however, its deficiency on macrophages may be responsible for the inhibition of phagocytosis and intracellular killing and the observed deficit of an antimycobacterial immunity. Among children with a clinical syndrome of IFN- γ–receptor deficiency, a clear genetic defect was identified in ≈20%. In our patient, the diagnosis was made by detection by flow cytometry of abnormally low expression of the α chain of the IFN-γ receptor on peripheral blood lymphocytes. This method appears to have high diagnostic value, given the fact that genetic methods are not always available and are expensive and often insensitive.

The prognosis in patients with BCG infection secondary to IFN-γ–receptor deficiency is unfavorable. A few cases of successful treatment with allogenic bone marrow transplantation have been reported with long-term improvement of general condition and stable receipt of the graft as shown by molecular analysis of peripheral leukocytes (*4**,**6**–**8*). However, as specific and efficient therapy for this condition has not been as yet proposed, supportive measures with early diagnosis and institution of anti-TB and antimicrobial drug treatment appear to be important in managing this rare immune deficiency. The level of IFN-γ-receptor expression in populations known to be susceptible to TB, and its potential role in this phenomenon, appears to be a promising area of study.
